# A multi-locus inference of the evolutionary diversification of extant flamingos (Phoenicopteridae)

**DOI:** 10.1186/1471-2148-14-36

**Published:** 2014-03-01

**Authors:** Chris R Torres, Lisa M Ogawa, Mark AF Gillingham, Brittney Ferrari, Marcel van Tuinen

**Affiliations:** 1 Department of Biology and Marine Biology, University of North Carolina Wilmington, Wilmington, North Carolina, USA; 2 Université de Bourgogne, Equipe Ecologie Evolutive, UMR CNRS 6282 Biogéosciences, 6 bd. Gabriel, Dijon 21000, France; 3 Centre de Recherche de la Tour du Valat, Le Sambuc, Arles 13200, France; 4 Leibniz Institute for Zoo and Wildlife Research, Department of Evolutionary Genetics, Alfred-Kowalke-Str. 17, Berlin D-10315, Germany

**Keywords:** Flamingo, Phylogeny, Divergence time, Biogeography, Fossil, Bill, Filter feeding

## Abstract

**Background:**

Modern flamingos (Phoenicopteridae) occupy a highly specialized ecology unique among birds and represent a potentially powerful model system for informing the mechanisms by which a lineage of birds adapts and radiates. However, despite a rich fossil record and well-studied feeding morphology, molecular investigations of the evolutionary progression among modern flamingos have been limited. Here, using three mitochondrial (mtDNA) markers, we present the first DNA sequence-based study of population genetic variation in the widely distributed Chilean Flamingo and, using two mtDNA and 10 nuclear (nDNA) markers, recover the species tree and divergence time estimates for the six extant species of flamingos. Phylogenetic analyses include likelihood and Bayesian frameworks and account for potential gene tree discordance. Analyses of divergence times are fossil calibrated at the divergence of Mirandornithes (flamingos + grebes) and the divergence of crown grebes.

**Results:**

mtDNA sequences confirmed the presence of a single metapopulation represented by two minimally varying mtDNA barcodes in Chilean flamingos. Likelihood and Bayesian methods recovered identical phylogenies with flamingos falling into shallow-keeled (comprising the Greater, American and Chilean Flamingos) and deep-keeled (comprising the Lesser, Andean and James’s Flamingos) sub-clades. The initial divergence among flamingos occurred at or shortly after the Mio-Pliocene boundary (6–3 Ma) followed by quick consecutive divergences throughout the Plio-Pleistocene. There is significant incongruence between the ages recovered by the mtDNA and nDNA datasets, likely due to mutational saturation occurring in the mtDNA loci.

**Conclusion:**

The finding of a single metapopulation in the widespread Chilean Flamingo confirms similar findings in other widespread flamingo species. The robust species phylogeny is congruent with previous classifications of flamingos based on feeding morphology. Modern phoenicopterids likely originated in the New World with each sub-clade dispersing across the Atlantic at least once. Our divergence time estimates place flamingos among the youngest families of birds, counter to the classical notion of flamingos as among the oldest based on biogeography and the fossil record. Finally, we designate ‘*Phoeniconaias*’ as a junior synonym of ‘*Phoenicoparrus*’ and redefine the latter genus as containing all flamingos more closely related to *Phoenicoparrus andinus* than *Phoenicopterus roseus*.

## Background

Flamingos are a unique order (Phoenicopteriformes) of birds with a highly specialized ecology but their evolutionary history remains poorly understood and until recently has only been informed by the fossil record. Flamingos are traditionally perceived as among the oldest lineages of living birds with reports of flamingo-like birds appearing in the fossil record as early as the late Cretaceous (*e.g.*[1]). However, the earliest birds reliably placed as stem phoenicopteriforms (family Palaelodidae) first appear in the early Oligocene of Europe [2] and the earliest members of the crown family (Phoenicopteridae) appear during the Oligo-Miocene of the Old World [3-5], suggesting an age for the family on par with most other major familial divergences within Aves [6]. Notably, *Harrisonavis croizeti,* an apparently morphologically modern flamingo from the Oligo-Miocene of France, suggests the modern flamingo divergence occurred in the Old World as early as the late Paleogene [3].

Recent fossil and molecular work have shed new insight into phoenicopterid origins and cast doubt on the classical notion of flamingos as a particularly ancient lineage among the storks, herons and ibises (Ciconiiformes). Most notably, molecular [7-11] and morphological [12,13] studies have supported a sister relationship between flamingos and grebes (Podicipediformes) as the clade Mirandornithes [14]. However, despite these advances, the exact age and phylogeny of modern flamingos remains to be robustly tested and several questions about flamingo evolution are in need of further investigation: (1) how do the six extant species of flamingos relate to each other, (2) how long ago did these divergences occur, and (3) where did crown Phoenicopteridae originate?

Sibley and Ahlquist [15] represents the only genetic study that addresses these questions. This study, based on differences in hybridization strength between genomic DNA of different species, documented a shallow age among five species and (the formerly classified) two subspecies (*Phoenicopterus ruber ruber* and *P. ruber roseus*) that likely form a single genus comprising two distinct sub-clades. This division is congruent with the organization by Jenkin [16] of flamingos into two groups reflecting mandibular morphology and feeding strategy: the Lesser, Andean and James’s Flamingos (the so-called deep-keeled group) have bulbous bills in cross-section suited to filtration of smaller food items (*e.g.* blue-green algae and diatoms); the remaining species (shallow-keeled group) have more compact bills in cross-section suited to filtration of larger food items (*e.g.* mollusks and crustaceans). Further morphological variation within the two sub-clades is not completely known [17].

Intraspecific genomic variation in each flamingo species is also incompletely known but is an important consideration when investigating morphological and geographic origins of species with wide ranges. Recent population genetics studies have identified a lack of genetic structure within the Greater [18]; Remi Wattier, *pers. comm.* and Lesser [19] Flamingos, both species with extensive ranges in the Old World. It is unlikely that the restricted breeding distribution [20,21] on the Andean altiplano promotes genetic structure in the Andean and James’s Flamingos. Except for captive populations [22,23], the population genetics of the American and Chilean Flamingos remains uninvestigated.

The present study has three aims: 1) to assess intraspecific mitochondrial variation within the widespread New World Chilean Flamingo through the use of three mtDNA markers and museum skins collected throughout the range; 2) to test the phylogenetic relationships among modern flamingos through the use of a multi-locus (10 nDNA and two mtDNA markers) and varied analytical (Bayesian, maximum likelihood framework) approach; and 3) to estimate the divergence times of living flamingo species from the resulting species tree calibrated with two fossil constraints. How these results inform the taxonomic organization and biogeography of flamingos is discussed.

## Results

### Population genetics of the Chilean Flamingo

Sequence data for 3 mtDNA loci were obtained and analyzed from 17–26 Chilean Flamingos representing the entire breeding range of the species except Ecuador (Additional file 1). Only two out of 18 individuals showed variation in the COI gene (one out of 142 bp, or 0.7%), exposing the same substitution recovered previously by a DNA barcoding study of neotropical birds [24]. That study indicates the presence of only one substitution (0.15%) in the entire barcode portion of the COI. From the current study, the common haplotype was distributed throughout the range, while the minor variant was found in an individual from Peru (this study) and in three individuals from the Argentinean Andes (this study and [24]). Both haplotypes coexist in these localities. Two additional markers that are known to be variable in related species (NADH 2, n = 17; control region, n = 20) failed to reveal the existence of additional mitochondrial variation.

### Phylogenetics

**Figure 1:**
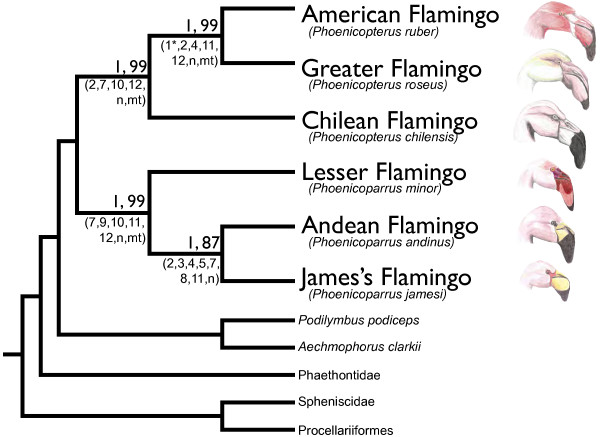
**Relationships and ages of the flamingos.** Cladogram showing the relationships of the living flamingos. The flamingos fall out in two sub-clades with high support, congruent with the topology recovered by Sibley and Ahlquist [15]. Genus *Phoenicoparrus* has been expanded to include all members of the deep-keeled clade (see Discussion). Posterior probabilities (Bayesian inference) and bootstrap support values (maximum likelihood) are shown above each node. Numbers and letters to the right of each node indicate individual genes and data subsets which recover that monophyletic clade. Key: 1, RHEB1; 2, TIMM17A; 3, TCF3; 4, RPS24; 5, SLC29A4; 6, NFKBIZ; 7, G3PDH; 8, myoglobin; 9, ZENK; 10, ZENK 3’UTR; 11, COI; 12, cyt b; n, nDNA subset; mt, mtDNA subset. RHEB1 failed to recover sister status between the American and Greater Flamingos in a maximum likelihood framework with high support, denoted by ‘1*’.

Analysis of single loci generally failed to result in complete phylogenetic resolution, instead supporting one or few of the key nodes in the final species tree (Figure 1). None of the single locus-based gene trees (except cyt b) indicate significant conflict, rather lack of resolution, with the final multi locus-based species tree. Thus, the joint analyses of loci that vary in mutation rate contributed to enhanced overall resolution The species topology is congruent with previous non-DNA sequence based studies [15] with flamingos breaking into two distinct clades: a shallow-keeled clade comprising (Chilean + (American + Greater)) and a deep-keeled clade comprising (Lesser + (Andean + James’s)).

Additional analysis indicates that the species topology is particularly robust. The Bayes factor analysis supported partitioning of the total dataset by locus (Bayes factor = 231), though both partitioned and non-partitioned BI analyses recovered the same topology with complete support (posterior probability = 1.0); the ML analysis had >85% bootstrap support for all nodes. A *BEAST analysis including multiple individuals for each flamingo species again recovered the same topology with complete support (posterior probability = 1.0). All jackknife analyses excluding a single locus at a time from the total (nDNA + mtDNA) dataset recovered the complete topology with high (>0.9) posterior probability, suggesting that no single locus was driving the results. The nDNA dataset recovered the same topology, while the mtDNA dataset differed only by recovering a (Andean + (Lesser + James’s)) clade, albeit without significant support.

Even though intra-specific variation was discovered in a set of five nDNA loci including two to three individuals per flamingo species, this variation was found to be minimal (<0.5% in each case, Additional file 2), an inference confirmed by independent cross-checking between the two genetics labs which revealed a match for all 6 flamingos. Furthermore, five of the 12 loci showed interspecific variation between the American and Greater Flamingos (0.1-1.8%; Additional file 3), with the barcode locus (COI) K2P distance indicating 1% variation.

### Divergence time estimation

The dates recovered by the mtDNA dataset are consistently ≥2 times the ages recovered by the nDNA dataset, while those recovered by the total dataset fall in between (Table 1). Crown-clade Phoenicopteridae likely arose at or shortly after the Mio-Pliocene boundary (3.0-6.5 mya). The deep- and shallow-keeled clades diverged in either the Pliocene or earliest Pleistocene (1.7-3.9 mya) followed by the American-Greater split (0.9-1.5 mya) and the Andean-James’s split (0.5-2.5 mya). Age ranges represent the total (upper bound) and the nDNA only (lower bound) datasets. See Table 1 for divergence dating results of the all datasets (ages in bold, 95% confidence intervals in parentheses).

**Table 1:** Ages for divergences within Phoenicopteridae based on 12 loci with 2 fossil calibrations

	**Age (95% C.I.) Ma**				
**Divergence**	**Total**	**Nuclear**	**Mitochondrial**	**Nuclear + COI**	**Nuclear + cyt b**
Crown	**4.37** (2.38-7.16)	**3** (1.45-5.5)	**6.5** (3.65-10.15)	**4.02** (2.24-6.73)	**5.59** (3.08-9.66)
*Phoenicopterus*	**2.29** (1.07-4.06)	**1.67** (0.69-3.21)	**2.42** (1.12-4.22)	**2.12** (0.99-3.67)	**2.42** (1.08-4.51)
*Ruber*-*roseus*	**1.01** (0.34-1.98)	**0.88** (0.25-1.87)	**1.18** (0.45-2.2)	**0.95** (0.34-1.83)	**1.45** (0.51-2.93)
*Phoenicoparrus*	**2.56** (1.26-4.37)	**1.83** (0.77-3.5)	**4.3** (2.15-6.9)	**2.08** (1.06-3.64)	**3.82** (1.96-6.69)
*Andinus*-*jamesi*	**1.34** (0.54-2.47)	**0.5** (0.12-1.14)	Not recovered	**0.81** (0.33-1.56)	**2.32** (1.07-4.23)

Ages of divergences within flamingos are in bold followed by the 95% confidence intervals in parentheses. Extant flamingos likely originated at or shortly after the Miocene-Pliocene boundary, making them one of the youngest lineages among living birds. The ages recovered by the nuclear data are consistently much younger than the ages recovered by the mitochondrial data, likely the result of mutational saturation in the mitochondrial sequences.

## Discussion

### Population genetics of the Chilean Flamingo

A single metapopulation was identified across the range of the Chilean Flamingo, harboring minimal genetic variation. This finding parallels similar recent findings in other widespread flamingo species [18,19] and is consistent with the appearance of opportunistic breeding [25] and a nomadic lifestyle [26] in all flamingos. Given these results, minimal genetic structure is expected for the phylogeographically unstudied Andean, James’s and American Flamingos. Thus, it is unlikely that unaccounted-for population structure impacted the results of our phylogenetic and divergence dating analyses.

### Phylogenetics

Our study provides the first sequence-based molecular support for the relationships among flamingos suggested previously [15,16]. By contrasting the lack of full resolution for any of the individual loci with the complete resolution found for the nDNA and total datasets, the need for multiple independent loci to resolve temporally young divergences is underscored. Conflicting phylogenetic signal exists between the mtDNA loci as COI was the only single locus to fully resolve the flamingo relationships while cyt b recovered the Andean Flamingo at the base of the deep-keeled clade with high support. The relationships recovered by the total dataset agreed with those recovered by COI, as well as by the nDNA dataset and those recovered previously [15], indicating that the nDNA data sufficiently overpowered the conflicting cyt b signal.

The two sub-clades recovered in our analysis are congruent with the patterns detected by Jenkin [16] in mandibular morphology. The shallow-keeled clade comprises those species with bills suited to capture larger prey items and includes the Greater (*Phoenicopterus roseus*), American (*P. ruber*) and Chilean (*P. chilensis*) Flamingos. Within this clade, the American and Greater Flamingos are often considered sub-species (*e.g.*[15]). The 1% divergence inferred from the DNA barcode locus falls in the range of expected nearest neighbor distances among sister species, such as seen commonly in waterfowl [27]. Although more sampling is needed to confirm species status, our finding of fixed mutational differences in several nuclear loci and a 1% divergence in mtDNA is consistent with treating the American and Greater Flamingos as separate species.

The deep-keeled clade comprises those species with bills suited to capturing smaller prey items and includes the Lesser (*minor*), Andean (*andinus*) and James’s (*jamesi*) Flamingos. These species have often been set apart from the classical genus *Phoenicopterus* with the Lesser placed within *Phoeniconaias* based on unique characteristics of the bill [28] and the Andean and James’s placed together in *Phoenicoparrus* based on the shared absence of the hallux (hind toe) [29]. However, the morphological definition of *Phoeniconaias* does not seem to exclude *andinus* and *jamesi*, and the hallux of *minor* is reduced [16], approximating the morphological definition of *Phoenicoparrus*. Thus, we suggest that the division of the deep-keeled clade into two genera is arbitrary and uninformative. Instead, we suggest the placement of *minor* within *Phoenicoparrus* along with *andinus* and *jamesi* based on shared mandibular morphology, ecology and phylogenetic relatedness. We suggest the designation of *Phoeniconaias* as a junior synonym of *Phoenicoparrus* based on priority and the redefinition of *Phoenicoparrus* as all species more closely related to *Phoenicoparrus andinus* than to *Phoenicopterus roseus*.

### Divergence time estimation

Even though the various subsets of data recovered incongruent divergence times (Table 1), all agreed on a recent divergence for crown Phoenicopteridae, indicating that living flamingos are among the youngest lineages within Neoaves. This young age is counter to the general perception of the Phoenicopteridae as among the oldest lineages of living birds based on fossil data (*e.g.*[1]) but is congruent with the DNA-DNA hybridization data of Sibley and Ahlquist [15], which identified the close genetic relatedness of the flamingos and predicted an age of 5–6 Ma for the basal divergence among the extant species. The nDNA dataset recovered ages significantly (≥2×) younger than those of the mtDNA dataset. This pattern is likely due in part to artificial signal caused by saturation of the more quickly mutating mtDNA genes [30]. Though saturation is unlikely to be an issue with divergences as shallow as those of crown Phoenicopteridae, the use of temporally more distant calibrations external to the clade of interest (*e.g.* within grebes) may have introduced saturation artifact. It is likely that homoplasy within the relatively longer and more quickly-evolving mtDNA sequences, with more mutational sites, conflicted with the signal of the shorter and more slowly evolving nDNA sequences, resulting in artificially old age estimates. Thus, the true ages of the component flamingo divergences are probably somewhere between those predicted by the total dataset and by the nDNA dataset alone.

### Biogeography

The two Old World species (Greater and Lesser) are each recovered in clades with otherwise exclusively New World distributions. The recovery of the Greater Flamingo as derived within *Phoenicopterus* suggests a New World origin for the shallow-keeled flamingos. The origin of *Phoenicoparrus* is not so straightforward, as the basal divergence within the clade spans the Atlantic. Plio-Pleistocene fossil flamingos are known only from the New World [31-37] and Australia [4,38] and the phylogenetic affinities of these species are uncertain. No flamingos occupy Australia today and it is unknown if the Plio-Pleistocene fossils represent an extension of the current distribution or if they are descendants of the species which occupied Australia in the Oligo-Miocene [4]. The New World fossil record, however, is congruent with the modern flamingo distribution and likely suggests an origin for *Phoenicoparrus*, and crown Phoenicopteridae, in the Western Hemisphere, followed by trans-Atlantic dispersal. Better phylogenetic resolution for the Australian forms will further inform phoenicopterid origins.

### Implications for the fossil record

The phoenicopteriform fossil record is rich but poorly understood and revision will greatly advise divergence time estimation and paleo-biogeographical reconstruction for crown flamingos. The stem phoenicopteriform record is characterized by fragmentary material with only tentative assignment to the flamingos. While the earliest palaelodids (early Oligocene) are the earliest to be reliably placed in Phoenicopteriformes, *Juncitarsus*[39,40] (but also see [41]), *Elornis* and *Agnopterus*[42] from the middle to late Eocene of North America and Europe may be earlier members and perhaps more appropriate fossil calibrations for Mirandornithes. Better understanding of the Plio-Pleistocene record may also provide internal calibrations for crown flamingos.

Finally, while we present the first information on the timing of diversification and specialization within crown flamingos, better understanding of interspecific variation (particularly with respect to the filter feeding mandibular morphology) is necessary to interpret the ecological significance of these results. Likewise, revision of the stem phoenicopteriform fossil record is necessary to place these ages in a more meaningful context. Most notably, the primitive flamingo *Harrisonavis croizeti* from the Oligo-Miocene of Europe displayed a mandibular morphology approximating that of modern flamingos [3,43]. Reassessment of this material, and new descriptions of contemporaneous taxa, will inform the state of flamingo specialization at this point and better constrain the rate of specialization within the Phoenicopteridae.

## Conclusions

Chilean Flamingos show no evidence of population structure across their entire range, a condition similar to other widely-distributed flamingo species. The six living species of flamingos fall into two clades reflecting differences in bill morphology and feeding ecology. We propose slight reorganization of flamingo taxonomy to indicate these differences: the Greater, American and Chilean Flamingos comprise genus *Phoenicopterus* while the Lesser is moved to genus *Phoenicoparrus* (= ‘*Phoeniconaias*’) along with the James’s and Andean Flamingos. The living species diverged from each other ~4.37 million years ago, followed by *Phoenicoparrus* (~2.56 mya) and *Phoenicopterus* (~2.29 mya).

## Methods and materials

### Population genetics methods

Field sampling of all flamingo populations remains impractical for logistic reasons, including geographic inaccessibility and conservation protection. Sampling from captive birds alone is not guaranteed to capture species-wide variation due to a persistent lack of information regarding the geographic origins of the ancestors of captive populations. To assess phylogeographic structure, we thus set out to sample museum specimens of Chilean Flamingos with known locality. Available DNA barcodes from Chilean Flamingos suggest the presence of at least two haplotypes in Argentina [19] but no data is available from other portions of their range. Thus, we examined portions of three mitochondrial genes through amplification from historic toe pads from Chilean Flamingos (n = 27) with known localities (see Additional file 1 for specimen information). The samples used spanned nearly the entire range known of Chilean Flamingos (excluding Ecuador) and were obtained through several museum loans. The three loci were chosen based on known intraspecific variation in flamingos (COI in Chilean and Greater Flamingos; NADH 2 in Lesser Flamingos) or known high rates of mutation (control region). Primers were manually generated using sequences available in GenBank (Additional file 4). In order to prevent contamination, ancient DNA procedures for extraction and amplification were followed [44] (see Additional file 5 for methods).

### Taxonomic sampling (phylogenetics)

Tissue samples were obtained for each flamingo species (Additional files 6, 7). *Podilymbus podiceps* and *Aechmophorus clarkii* were included to represent the basal split within grebes [45]. The sister taxon to (flamingos + grebes) remains controversial but the tropicbirds (Phaethontidae) are consistently found as close relatives (*eg.*[7,9,10]) and were used as an outgroup. Penguins (Sphenisciformes) and tubenoses (Procellariiformes) were included as representatives of the waterbird clade (*eg.*[9-11]). Sample collection was reviewed and approved by the Centre de Recherche sur la Biologie des Populations d’Oiseaux (CRBPO, Musée National d’Histoire 126 Naturelle, France) under the personal permit (number 405) of Alan Johnson and Arnaud Béchet and by USDA permit (number 102976) of MVT.

### Genomic sampling

Genomic DNA was extracted from tissue samples using the DNeasy Tissue Kit (Qiagen) and was amplified for 10 nuclear and two mitochondrial loci from one individual per species, which comprised the primary dataset (Table 2). Two additional nuclear loci were also amplified to test intraspecific variation among flamingos only. See Additional file 4 for primer sequences. The nuclear loci consisted primarily of intronic regions with primers designed using an exon-primed intron spanning approach and sequences from the chicken and zebra finch genome (following [46]). Polymerase chain-reaction (PCR) amplifications were carried out in 50 µl reactions comprising: 31 µl dH_2_O, 5 µl 10× detergent- and Mg^+^-free reaction buffer, 5 µl MgCl_2_ (25 mM), 1 µl dNTP (10 mM each), 2.6 µl each primer, 0.2 µl Hot Multi*Taq* (US DNA) polymerase (5 u/µl) and 2.5 µl DNA template (20 mM). Thermocycling comprised an initial denaturation at 95°C for 5 min followed by 35–40 cycles of annealing at 95°C for 30 sec, extension at 50°C for 30 sec and denaturation at 95°C for 40 sec, followed by a final annealing/extension at 72°C for 5 min. Excess dNTPs and primers were removed from PCR product by addition of 1.45 µl Exonuclease 1 (Fermentas; 5 u/µl), 2.85 µl FastAP (Thermo Scientific) shrimp alkaline phosphatase (1 u/µl) and 2.85 µl dH20 and held at 37°C for 30 min and then 80°C for 15 min. Product was sequenced offsite at MacroGen USA (Maryland).

**Table 2:** Genetic loci used in the phylogenetic and divergence time estimation analyses

**Locus**	**Basepair length**	**Genomic location**	**Phylo. sub. model**	**Diverg. sub. model**
RHEB1	642	Nuclear	T92 + G	TN93 + I
TIMM17A	516	Nuclear	K2P	HKY
TCF3	601	Nuclear	T92	HKY
RPS24	407	Nuclear	HKY	HKY
SLC29A4	524	Nuclear	TN93 + I	TN93 + I
NFKBIZ	506	Nuclear	K2P	HKY
G3PDH	432	Nuclear	HKY	HKY
myoglobin	677	Nuclear	K2P	HKY
ZENK	653	Nuclear	TN93	TN93
ZENK 3’UTR	287	Nuclear	T92	HKY
COI	699	Mitochondrial	HKY + G	HKY + G
cyt b	1026	Mitochondrial	HKY + G	HKY + G
ADAMTS10	510	Nuclear	K2	--
HMGB2	575	Nuclear	T92 + I	--

Only the two mtDNA loci were treated as coding; the mtDNA were further partitioned by codon in the divergence time estimation analyses. Substitution models for each locus were estimated using MEGA 5.2 and the best available model was used in MEGA 5.2 and MrBayes 3.2.1 for the phylogenetic analyses and in BEAST 1.8.0 for the divergence time estimation analyses. ADAMTS10 and HMGB2 were only used in phylogenetic analysis testing flamingo intraspecific variation. Abbreviations: RHEB1, Rheb isoform 1, intron 3; TIMM17A, mitochondrial import inner membrane translocase subunit Tim17A, intron 3; TCF3, Transcription factor 3, intron 12; RPS24, ribosomal protein S24, intron 5; SLC29A4, solute carrier family 29, member 4, intron 8; NFKBIZ, Nuclear Factor of light polypeptide gene enhancer in B-cells inhibitor, zeta, intron 6; G3PDH, glyceraldehyde-3-phosphate dehydrogenase gene, intron 11; myoglobin gene, exon 2, 3 and intron 2; ZENK, zinc finger protein, exon 2; ZENK 3’UTR, zinc finger protein, 3’ untranslated region; COI, cytochrome oxidase subunit I; cyt b, cytochrome b; ADAMTS10, ADAM metallopeptidase with thrombospondin, type 1 motif, intron 5; HMGB2, high mobility protein group, box 2, intron 1. Drawings by M. McCracken.

Sequence data for one to two additional flamingo individuals per species for five loci (Additional file 8) were collected in the Dijon, France lab. For these samples, genomic DNA was extracted from blood samples for the Greater, American and Chilean Flamingos and feather samples for the remaining three species using a standard phenol-chloroform method. PCR amplifications and product clean-up followed the same protocols as the Wilmington, US lab. Product was sequenced offsite at MacroGen Korea (Seoul).

### Sequence treatment and phylogenetic analysis

Sequences were aligned within Sequencher 4.8 and alignments were confirmed within MEGA 5.2 [47] using ClustalW with default settings. Pairwise genetic distances were calculated in MEGA 5.2 using the default Maximum Composite Likelihood substitution model except for COI, which was calculated using Kimura 2-Parameter. Maximum likelihood phylogenetic analyses were carried out within MEGA 5.2 and the loci were concatenated. Unless the dataset comprised only coding genes (ZENK, COI and cyt b), each dataset was treated as noncoding; optimal substitution models were found within the hierarchical BI and ML framework in MEGA 5.2 (Table 2) and the analysis used a nearest-neighbor-interchange heuristic search. Bootstrap support was estimated from 500 replicates using the same settings. Primary phylogenetic analyses using Bayesian inference were carried out in MrBayes 3.2.1 [48]. Datasets were fully partitioned based on the optimal substitution models estimated within MEGA 5.2. Each search comprised two concurrent runs of four chains each for 10,000,000 generations sampled every 1,000 generations with the first 1,000,000 generations discarded as burn-in. The results of each analysis were tested for convergence of phylogenetic signal in Tracer 1.5 [49], where estimated sample size (ESS) values >200 were treated as reliable signal.

The primary analysis employed all 10 nDNA and two mtDNA loci for one individual per species. All secondary analyses employed the same parameters as the primary analysis except where noted. To test for the presence of intraspecific variation, a series of single-locus analyses were carried out using two to three sequences per flamingo species for five loci (Additional file 2). To recover individual gene trees, each locus was analyzed independently. To test the relative weight of each locus to the combined dataset, a series of analyses were run excluding a single gene at a time. To test for conflicting signal between the nDNA and mtDNA datasets, each was analyzed independently. To test for the effects of over-partitioning of the Bayesian inference analysis, a Bayesian analysis was carried out treating the total dataset as non-partitioned (as in the ML analysis). A Bayes factor analysis was carried out in Tracer 1.5 to compare the strength in phylogenetic signal of the fully partitioned and non-partitioned BI treatments.

*BEAST 1.8.0 [50] was used to test for discordance between individual gene trees and the species tree. The dataset was fully phased by individual and gene. Loci were allowed unlinked substitution models, clock models and partition trees. The optimal available substitution model was used (Table 2) and the 3 coding loci were partitioned by codon position. Each locus was allowed a lognormal relaxed clock with uncorrelated rates. Mean clock rate priors (ucld.mean) were allowed uninformative uniform distributions (Initial value = 0.05; Upper = 1.0E100; Lower = 0.001). A Yule speciation process was assumed. No fossil node calibrations were used. The results were tested for convergence of phylogenetic signal in Tracer 1.5.

### Divergence timing analysis and fossil calibrations

Divergence time estimation analyses were carried out using BEAST 1.8.0 [51]. Loci were allowed unlinked substitution models (Table 2) and the 3 coding loci were partitioned by codon position. A lognormal relaxed clock with uncorrelated rates was assumed and a Yule speciation process was employed. The clock mean (ucld.mean) was allowed an uninformative uniform prior distribution (Initial value = 0.05; Upper = 1.0E100; Lower = 0.001). Taxa were divided into sets representing grebes and Mirandornithes; monophyly of these sets was not enforced. Two nodes were fossil calibrated following the guidelines suggested by Parham et al. [52]. The divergence of extant grebes was calibrated at 8.7 mya based on *Thiornis sociata*, a grebe from the late Miocene of Spain [53,54]. The divergence of flamingos and grebes was calibrated at 32.6 mya based on *Adelalopus hoogbutseliensis*, a stem phoenicopteriform from the earliest Oligocene of Belgium [2]. Calibrations were allowed lognormal distributions with offset = calibration age and standard deviation = 1.0. See Table 1 for the data included in each divergence analysis. All analyses were run twice for 100 million generations sampled every 1000 with burn-in of 1 million. Convergence of phylogenetic signal was tested for in Tracer 1.5.

### Availability of supporting data

All supporting data are included as additional files. Newly obtained DNA sequence reads have been uploaded to Genbank (http://www.ncbi.nlm.nih.gov/). Genbank accession numbers are provided in Additional files 7 and 8.

## Competing interests

The authors declare that they have no competing interests.

## Authors’ contributions

CRT carried out DNA extraction, amplification, alignment, performed all phylogenetic and divergence time analysis, and drafted the manuscript. LMO and MAFG carried out DNA extraction, amplification, and alignment, and helped draft the manuscript. BF carried out ancient DNA extraction, amplification and alignment. MVT conceived of the study, participated in its design and coordination, assisted with alignment and analysis, and helped to draft the manuscript. All authors read and approved the final manuscript.

## Supplementary Material

Additional file 1 Specimen and locality information for the Chilean Flamingo individuals included in the population genetics analysis. Click here for file

Additional file 2 Pairwise genetic distances between multiple individuals of each flamingo species for five nDNA loci. Click here for file

Additional file 3 **Pairwise genetic distances between *Phoenicopterus ruber *** **and *P. roseus *** **for 10 nDNA and two mtDNA loci.** Click here for file

Additional file 4 Primer sequences for each locus used in this study. Click here for file

Additional file 5 Ancient DNA extraction and amplification methods. Click here for file

Additional file 6 Sample information for individuals used in the primary phylogenetic analyses. Click here for file

Additional file 7 GenBank accession numbers for sequence data used in the primary phylogenetic analyses. Click here for file

Additional file 8 Sample information for the additional flamingo individuals used in the pairwise genetic distance analyses and GenBank accession numbers for associated sequence data. Click here for file
