# Supplementary material for: A multi-locus inference of the evolutionary diversification of extant flamingos (Phoenicopteridae)
Source: BMC Evol Biol. 2014 Mar 1;14:36. doi: 10.1186/1471-2148-14-36 (PMC4016592; doi:10.1186/1471-2148-14-36)
Supplement: Additional file 1 — Specimen and locality information for the Chilean Flamingo individuals included in the population genetics analysis. [file 1471-2148-14-36-S1.doc]

Additional files

Additional file 1 – Specimen information for the Chilean Flamingo individuals included in the population genetics analysis. Sequence data available upon request.

| Sample source | Extraction No. | Location | Year |
| --- | --- | --- | --- |
| AMNH 445149 | Pch 6.1 | Ancud, Chile | 1914 |
| AMNH 469861 | Pch 6.2 | Sajama, Bolivia | 1901 |
| AMNH 445157 | Pch 6.3 | Junin, Peru | 1913 |
| AMNH 445162 | Pch 6.4 | Junin, Peru | 1913 |
| AMNH 469866 | VF 3.1 | Salado, Argentina | 1901 |
| AMNH 469864 | VF 3.2 | Buenos Aires, Argentina | 1897 |
| AMNH 269794 | VF 3.3 | Independence Bay, Peru | 1919 |
| AMNH 445154 | VF 3.4 | Pisco, Peru | 1913 |
| AMNH 445151 | VF 3.5 | Punta Arenas, Chile | 1914 |
| AMNH 445152 | VF 3.6 | Punta Arenas, Chile | 1914 |
| AMNH 445156 | VF 4.1 | Junin, Peru | 1913 |
| AMNH 445153 | VF 4.2 | St. Magellan, Chile | 1914 |
| AMNH 445158 | VF 4.3 | Punta Arenas, Chile | 1914 |
| AMNH 469865 | VF 4.4 | Buenos Aires, Argentina | 1901 |
| AMNH 469863 | Pch 4.1 | Buenos Aires, Argentina | 1897 |
| AMNH 445155 | Pch 4.2 | Lake Titicaca | 1913 |
| AMNH 445160 | Pch 4.7 | Junin, Peru | 1913 |
| LSUMZ B61032 | Pch 5.1 | Cochabamba, Bolivia | *n/a* |
| LSUMZ B61034 | Pch 5.2 | Puno, Peru | *n/a* |
| LSUMZ B61033 | Pch 5.3 | Esperanza, Bolivia | *n/a* |
| ZB 16168 | Pch 7.2 | Mendoza, Argentina | 1925 |
| ZB 16170 | Pch 7.3 | Mendoza, Argentina | 1925 |
| ZB 18541 | Pch 7.4 | Mendoza, Argentina | 1925 |
| KU 9846 | VF 2.1 | Jujuy, Argentina | 2005 |
| CU 5774 | VF 1.1 | Lake Titicaca | 1938 |
| CU 9358 | VF 1.2 | Puno, Peru | 1938 |
