# Supplementary material for: A multi-locus inference of the evolutionary diversification of extant flamingos (Phoenicopteridae)
Source: BMC Evol Biol. 2014 Mar 1;14:36. doi: 10.1186/1471-2148-14-36 (PMC4016592; doi:10.1186/1471-2148-14-36)
Supplement: Additional file 2 — Pairwise genetic distances between multiple individuals of each flamingo species for five nDNA loci. [file 1471-2148-14-36-S2.doc]

Additional file 2 - Pairwise genetic distances between multiple individuals of the same species, by locus. Distances were estimated in MEGA 5.2 using the Kimura 2-parameter distances model. Two loci (TIMM17A and NFKBIZ) showed no intraspecific variation. The three remaining loci showed >0.5% intraspecific variation. TIMM17A, SLC29A4 and NFKBIZ were included in the primary phylogenetic analyses. ADAMTS10 and HMGB2 were used only to assess intraspecific variation and confirm correct species identifications. Sequence data for one individual each were collected in the Wilmington, NC lab and only these data were used in the primary analyses (except the *BEAST analysis; see Methods and Materials of the main text). Sequence data for all other individuals were collected in the Dijon, France lab. Dashes (–) denote unsuccessful amplifications.

| Taxon | Locus | TIMM17A | SLC29A4 | NFKBIZ | ADAMTS10 | HMGB2 |
| --- | --- | --- | --- | --- | --- | --- |
| *Phoenicopterus ruber* | length | 516 | 523 | 506 | 510 | 575 |
|  | variable sites | 0 | 0 | – | – | 1 |
|  | % variation | 0.0% | 0.0% | – | – | 0.2% |
|  | # individuals | 2 | 2 | 1 | 1 | 2 |
| *Phoenicopterus roseus* | length | 516 | 523 | 506 | 510 | 575 |
|  | variable sites | 0 | 2 | 0 | – | 1 |
|  | % variation | 0.0% | 0.4% | 0.0% | – | 0.2% |
|  | # individuals | 2 | 2 | 2 | 1 | 2 |
| *Phoenicopterus chilensis* | length | 516 | 523 | 506 | 510 | 575 |
|  | variable sites | 0 | 0 | 0 | 0 | 0 |
|  | % variation | 0.0% | 0.0% | 0.0% | 0.0% | 0.0% |
|  | # individuals | 2 | 2 | 2 | 2 | 2 |
| *Phoenicoparrus minor* | length | 516 | 523 | 506 | 510 | 575 |
|  | variable sites | 0 | 0 | 2 | 2 | 0 |
|  | % variation | 0.0% | 0.0% | 0.0% | 0.4% | 0.0% |
|  | # individuals | 2 | 3 | 3 | 2 | 3 |
| *Phoenicoparrus andinus* | length | 516 | 523 | 506 | 510 | 575 |
|  | variable sites | 0 | – | 0 | 0 | 0 |
|  | % variation | 0.0% | – | 0.0% | 0.0% | 0.0% |
|  | # individuals | 2 | 1 | 2 | 2 | 2 |
| *Phoenicoparrus jamesi* | length | 516 | 523 | 506 | 510 | 575 |
|  | variable sites | 0 | 1 | 0 | 0 | 0 |
|  | % variation | 0.0% | 0.2% | 0.0% | 0.0% | 0.0% |
|  | # individuals | 2 | 2 | 2 | 2 | 2 |
