# Supplementary material for: A multi-locus inference of the evolutionary diversification of extant flamingos (Phoenicopteridae)
Source: BMC Evol Biol. 2014 Mar 1;14:36. doi: 10.1186/1471-2148-14-36 (PMC4016592; doi:10.1186/1471-2148-14-36)
Supplement: Additional file 3 — Pairwise genetic distances between Phoenicopterus ruber and P. roseus for 10 nDNA and two mtDNA loci. [file 1471-2148-14-36-S3.doc]

Additional file 3 – Pairwise genetic distances between *Phoenicopterus ruber* and *P. roseus*, by locus. Distances were estimated in MEGA 5.2 using the Kimura 2-parameter distances model. Only 5 loci show variation and all are ≤1.8% variant (including COI).

| Locus | RHEB1 | TIMM17A | TCF3 | RPS24 | SLC29A4 | NFKBIZ | G3PDH | myoglobin | ZENK | ZENK 3’UTR | COI | cyt b | nDNA | mtDNA | Total |
| --- | --- | --- | --- | --- | --- | --- | --- | --- | --- | --- | --- | --- | --- | --- | --- |
| length | 642 | 516 | 601 | 407 | 524 | 506 | 432 | 677 | 653 | 287 | 699 | 1026 | 5245 | 1725 | 6970 |
| # diff | 2 | 0 | 0 | 0 | 0 | 0 | 7 | 1 | 0 | 0 | 7 | 15 | 10 | 22 | 32 |
| % diff | 0.30% | 0.00% | 0.00% | 0.00% | 0.00% | 0.00% | 1.80% | 0.10% | 0.00% | 0.00% | 1.00% | 1.50% | 0.20% | 1.30% | 0.50% |
