# Supplementary material for: A multi-locus inference of the evolutionary diversification of extant flamingos (Phoenicopteridae)
Source: BMC Evol Biol. 2014 Mar 1;14:36. doi: 10.1186/1471-2148-14-36 (PMC4016592; doi:10.1186/1471-2148-14-36)
Supplement: Additional file 4 — Primer sequences for each locus used in this study. [file 1471-2148-14-36-S4.doc]

Additional file 4 – Primer sequences used in this study. Asterisk (*) denotes primers used exclusively for Chilean Flamingo population genetics. Numbers in superscript indicate references (below) for primers not developed for this study.

| Locus | Forward Sequence | Reverse Sequence |
| --- | --- | --- |
| RHEB1 | ATACCCATTATGCTGGTTGG | AAAGCTGCATTCCAGGATTC |
| TIMM17A | ATCACAAGCGGAGCCCTGAC | GATCCAACCATGGCAACAGG |
| TCF3 | GTGCCTTATCTCCCAGCTACG | CCTCAGCACGTGGATGGCTTC |
| RPS24 | GACTTCTGCAGCGCAAGCAG | CAGGATGAAGAACATCGATC |
| SLC29A4 | GGTASACRCACATGATGAAGAGRG | GSTSATCTGGGCCTACATGC |
| NFKBIZ | CTGCCAAYCAGCATCTCATTG | GTTGCC TCAAGRTCCACAWAYTG |
| G3PDH^1^ | GGCATTGCACTGARYGAYCATTT | ARRTCCACAACACGGTTGCTGTA |
| Myoglobin^2^ | CAAATATCTRGAGGTAYGRAA | CAGAAATGAACTGTGAGGARRG |
| ZENK^3^ | AGAAACCAGCTATCCCAAYCAA | CGTGAAAACCTCCGGTCACAG |
| ZENK 3’UTR^3^ | CCACCTCTGTTGAATGACTTATTTGC | CTCATCAGGTGATATTTACAWAGCAT |
| COI | TTCTCAACCAACCACAAAGAYATYGG | ACGTGGGAGATGCCGAAKCCTG |
| cyt b^4^ | AAYATYTCWGYHTGATGAAAYTTYGG | CTTCANTYTTTGGYTTACAAGRCC |
| ADAMTS10 | CACCACCTTCCACTACAGGAG | GCGTTGAACTTGTAGCGGATC |
| HMGB2^5^ | GAAATGTGGTCTGAACAGTC | TTGCTCTTGGCACGATATGC |
| COI* | CACTAGCTGGCAACATAGC | GTGATGAGGACRGATCATACG |
| NADH 2* | CAGCCACCGTATTCCTCAC | GTGGCTGTTGTGGTTATTTCTTG |
| Control region* | CGGACCAGGTTATTTATTAATCG | GCGATCACGGACGRAAATG |

References

1. Van Tuinen M, Butvill DB, Kirsch JAW, Hedges SB: **Convergence and divergence in the evolution of aquatic birds**. *Proceedings of the Royal Society of London B* 2001, **268**:1345–1350.
2. Irestedt M, Fjeldsa J, Johansson US, Ericson PGP: **Systematic relationships and biogeography of the tracheophone suboscines (Aves: Passeriformes)**. *Molecular Phylogenetics and Evolution* 2002, **23**:499–512.
3. Chubb AL: **New nuclear evidence for the oldest divergence among neognath birds: the phylogenetic utility of ZENK (i)**. *Molecular Phylogenetics and Evolution* 2004, **30**:140–151.
4. Sorenson MD, Ast JC, Dimcheff DE, Yuri T, Mindell DP: **Primers for a PCR-based approach to mitochondrial genome sequencing in birds and other vertebrates**. *Molecular Phylogenetics and Evolution* 1999, **12**:105–114.
5. Jackson DG, Emslie SD, van Tuinen M: **Genome skimming identifies polymorphism in tern populations and species**. *BMC Research Notes* 2012, **5**.
