# Supplementary material for: A multi-locus inference of the evolutionary diversification of extant flamingos (Phoenicopteridae)
Source: BMC Evol Biol. 2014 Mar 1;14:36. doi: 10.1186/1471-2148-14-36 (PMC4016592; doi:10.1186/1471-2148-14-36)
Supplement: Additional file 5 — Ancient DNA extraction and amplification methods. [file 1471-2148-14-36-S5.doc]

Additional file 5 – Ancient DNA Extraction and Amplification Methods

The historic DNA extractions took place in a sterile genetics lab with designated reagents and equipment. Restrictions to this lab were in place to reduce the flow of traffic and the lab counters were cleaned with bleach and all equipment and plastic ware was sterilized by UV light. Gloves were worn at all times when handling the DNA in order to avoid contamination. Furthermore, for each extraction and amplification, a negative control was used to test for contamination. Due to risk of contamination, positive controls were not included. All obtained sequences were verified through two independent amplifications and sequence reactions. Variation in the COI gene was additionally verified through two separate extractions.

10mg of toe pad was cut in small pieces with a razor and lysis was then performed through a base-acid procedure. This procedure involved submerging the tissue in 75 µL of 1M NaOH for 90 minutes at 90 degrees Celsius, after which the samples were cooled off with ice and 75 µL of 20mM HCl was added. The supernatant was then removed by pipette and mixed with 75 µL of UltraClean Tissue and Cells DNA Isolation chaotropic buffer TD1 (Mo-Bio labs, Carlsbad, CA). The Power Clean DNA Cleanup procedure (Mo-Bio labs) was followed to extract the DNA and remove possible inhibitors.

A 12.5 µL pre-made Master mix solution (Taq2X Mastermix, New England Biolabs, Inc.) was used, to which 7.4 µL of distilled water, 2.5 µL of DNA template, 1.3 µL of forward primer (5uM) and 1.3 µL of reverse primer (5uM) were added. The tubes were then placed in a Peltier Thermal Cycler (PTC 225) and began with a 3 minute warm-up period at 95 degrees Celsius, and then followed by 42 repeats of the 3-part cycle of 30 seconds at 95 degrees Celsius for DNA denaturation, 30 seconds at 50 degrees Celsius for the DNA polymerase to anneal to the single strands of DNA, and 30 seconds at 72 degrees Celsius for the DNA polymerase to work. Afterwards, the samples were set at 72 degrees Celsius for seven minutes to finish replication.
