# Supplementary material for: A multi-locus inference of the evolutionary diversification of extant flamingos (Phoenicopteridae)
Source: BMC Evol Biol. 2014 Mar 1;14:36. doi: 10.1186/1471-2148-14-36 (PMC4016592; doi:10.1186/1471-2148-14-36)
Supplement: Additional file 6 — Sample information for individuals used in the primary phylogenetic analyses. [file 1471-2148-14-36-S6.doc]

Additional file 6 – Sample information for taxa used in the primary phylogenetic analyses. Numbers denote the loci for which each specimen was used and correspond to: 1, RHEB1; 2, TIMM17A; 3, TCF3; 4, RPS24; 5, SLC29A4; 6, NFKBIZ; 7, G3PDH; 8, myoglobin; 9, ZENK; 10, ZENK 3’UTR; 11, COI; 12, cyt b; 13, ADAMTS10; 14, HMGB2.

| Order | Family | Species | Common Name | Specimen Information |
| --- | --- | --- | --- | --- |
| Phoenicopteriformes | Phoenicopteridae |  |  |  |
|  |  | *Phoenicopterus ruber* | American Flamingo | Sibley and Ahlquist (1990)^1-6,8-10,12-14^ |
|  |  |  |  | Genbank (locality unknown)^7,11^ |
|  |  | *Phoenicopterus roseus* | Greater Flamingo | Camargue, France^1-14^ |
|  |  | *Phoenicopterus chilensis* | Chilean Flamingo | Jujuy, Argentina (KU 9846)^1-10, 12-14^ |
|  |  |  |  | Genbank (Catamarca, Argentina)^11^ |
|  |  | *Phoenicoparrus minor* | Lesser Flamingo | captive^1-14^ |
|  |  | *Phoenicoparrus andinus* | Andean Flamingo | Sibley and Ahlquist (1990)^1-10, 13-14^ |
|  |  |  |  | Genbank (Catamarca, Argentina^11^; unknown^12^) |
|  |  | *Phoenicoparrus jamesi* | James’s Flamingo | no locality data (AMNH DOT 17450)^1-11, 13-14^ |
|  |  |  |  | Potosi, Bolivia (UMMZ 156240)^12^ |
| Podicipediformes | Podicipedidae |  |  |  |
|  |  | *Podilymbus podiceps* | Pied-billed Grebe | Humboldt, California (PBGR STUY 280)^1,8^ |
|  |  |  |  | Raleigh, NC (UNCW B 1150)^2-5^ |
|  |  |  |  | New Mexico (NMSU 1995x)^6-7^ |
|  |  |  |  | Artigas, Uruguay (YPM 101067)^9-10^ |
|  |  |  |  | Genbank (Ontario, Canada^11^; unknown^12^) |
|  |  | *Aechmophorus clarkii* | Clark’s Grebe | San Francisco, CA (USNM B21500)^1-12^ |
| Phaethontiformes | Phaethontidae |  |  |  |
|  |  | *Phaethon rubricauda* | Red-tailed Tropicbird | Johnston Island, Pacific Ocean (UWBM 68951)^1-5,8-9,11^ |
|  |  |  |  | Genbank (Marshall Islands, Micronesia^10^; unknown^12^) |
|  |  | *Phaethon aethereus* | Red-billed Tropicbird | Ocracoke, NC (NMSU 20567)^6-7^ |
| Sphenisciformes | Spheniscidae |  |  |  |
|  |  | *Pygoscelis antarcticus* | Chinstrap Penguin | captive^1-7,11^ |
|  |  |  |  | Genbank (locality unknown)^12^ |
|  |  | *P. adeliae* | Adelie Penguin | Genbank (locality unknown)^9-10^ |
|  |  | *Spheniscus humboldti* | Humboldt Penguin | Genbank (locality unknown)^8^ |
| Procellariiformes | Hydrobatidae |  |  |  |
|  |  | *Oceanites oceanicus* | Wilson’s Storm Petrel | Carteret, NC (NCSM 22701)^1-3,5-7,9,12^ |
|  |  |  |  | Genbank (locality unknown^8,10^; Valparaiso, Chile^11^) |
|  | Procellariidae |  |  |  |
|  |  | *Macronectes giganteus* | Southern Giant Petrel | Palmer Station, Antarctic Peninsula (UNCW B 1131)^4^ |
