# Supplementary material for: A multi-locus inference of the evolutionary diversification of extant flamingos (Phoenicopteridae)
Source: BMC Evol Biol. 2014 Mar 1;14:36. doi: 10.1186/1471-2148-14-36 (PMC4016592; doi:10.1186/1471-2148-14-36)
Supplement: Additional file 7 — GenBank accession numbers for sequence data used in the primary phylogenetic analyses. [file 1471-2148-14-36-S7.doc]

Additional file 7 – Genbank accession numbers for sequences used in the primary phylogenetic analysis. Asterisks (*) denote sequences not collected in this study. All other sequences were collected by the Wilmington lab (see Additional file 8 for sequences collected by the Dijon lab). Dashes (–) denote sequences which were not included in this study.

| Taxon | RHEB1 | TIMM17A | TCF3 | RPS24 | SLC29A4 | NFKBIZ | G3PDH |
| --- | --- | --- | --- | --- | --- | --- | --- |
| *Phoenicopterus ruber* | KJ400204 | KJ400215 | KJ400232 | KJ400253 | KJ400264 | KJ400294 | AF339352* |
| *Phoenicopterus roseus* | KJ400205 | KJ400216 | KJ400233 | KJ400254 | KJ400265 | KJ400295 | KJ400324 |
| *Phoenicopterus chilensis* | KJ400206 | KJ400217 | KJ400234 | KJ400255 | KJ400266 | KJ400296 | KJ400325 |
| *Phoenicoparrus andinus* | KJ400207 | KJ400218 | KJ400235 | KJ400256 | KJ400267 | KJ400297 | KJ400326 |
| *Phoenicoparrus jamesi* | KJ400208 | KJ400219 | KJ400236 | KJ400257 | KJ400268 | KJ400298 | KJ400327 |
| *Phoenicoparrus minor* | KJ400209 | KJ400220 | KJ400237 | KJ400258 | KJ400269 | KJ400299 | KJ400328 |
| *Podilymbus podiceps* | KJ400210 | KJ400221 | KJ400238 | KJ400259 | KJ400270 | KJ400300 | KJ400329 |
| *Aechmophorous clarkii* | KJ400211 | KJ400222 | KJ400239 | KJ400260 | KJ400271 | KJ400301 | KJ400330 |
| Phaethontidae | KJ400212 | KJ400223 | KJ400240 | KJ400261 | KJ400272 | KJ400302 | KJ400331 |
| Spheniscidae | KJ400213 | KJ400224 | KJ400241 | KJ400262 | KJ400273 | KJ400303 | KJ400332 |
| Procellariiformes | KJ400214 | KJ400225 | KJ400242 | KJ400263 | KJ400274 | KJ400304 | KJ400333 |

| Taxon | myoglobin | ZENK | ZENK 3’UTR | COI | cyt b | ADAMS10 | HMGB2 |
| --- | --- | --- | --- | --- | --- | --- | --- |
| *Phoenicopterus ruber* | KJ400334 | KJ400342 | KJ400352 | JN801358* | KJ400317 | KJ400243 | KJ400281 |
| *Phoenicopterus roseus* | KJ400335 | KJ400343 | KJ400353 | KJ400311 | KJ400318 | KJ400244 | KJ400282 |
| *Phoenicopterus chilensis* | KJ400336 | KJ400344 | KJ400354 | FJ028027* | KJ400319 | KJ400245 | KJ400283 |
| *Phoenicoparrus andinus* | KJ400337 | KJ400345 | KJ400355 | FJ028025* | U08939* | KJ400246 | KJ400284 |
| *Phoenicoparrus jamesi* | KJ400338 | KJ400346 | KJ400356 | KJ400312 | KJ400320 | KJ400247 | KJ400285 |
| *Phoenicoparrus minor* | KJ400339 | KJ400347 | KJ400357 | KJ400313 | KJ400321 | KJ400248 | KJ400286 |
| *Podilymbus podiceps* | KJ400340 | KJ400348 | KJ400358 | DQ433969* | EU167015* | – | – |
| *Aechmophorous clarkii* | KJ400341 | KJ400349 | KJ400359 | KJ400314 | KJ400322 | – | – |
| Phaethontidae | DQ881864* | KJ400350 | EU738834* | KJ400315 | AP009043* | – | – |
| Spheniscidae | DQ881875* | AF490195* | AF490144* | KJ400316 | DQ137221* | – | – |
| Procellariiformes | EU739998* | KJ400351 | EU738823* | DQ433048* | KJ400323 | – | – |
