# Supplementary material for: A multi-locus inference of the evolutionary diversification of extant flamingos (Phoenicopteridae)
Source: BMC Evol Biol. 2014 Mar 1;14:36. doi: 10.1186/1471-2148-14-36 (PMC4016592; doi:10.1186/1471-2148-14-36)
Supplement: Additional file 8 — Sample information for the additional flamingo individuals used in the pairwise genetic distance analyses and GenBank accession numbers for associated sequence data. [file 1471-2148-14-36-S8.doc]

Additional file 8 – Sample information and Genbank accession numbers for additional flamingo individuals obtained by the Dijon lab. Sequences were included only in the *BEAST analysis and to evaluate intraspecific variation. Dashes (–) denote unsuccessful amplifications.

| Species | | Specimen Info | | TIMM17A | SLC29A4 | NFKBIZ | ADAMTS10 | HMGB2 |
| --- | --- | --- | --- | --- | --- | --- | --- | --- |
| *Phoenicopterus ruber* |  | | captive | KJ400226 | KJ400275 | – | – | KJ400287 |
| *Phoenicopterus roseus* | | Camargue, France | | KJ400227 | KJ400276 | KJ400305 | – | KJ400288 |
| *Phoenicopterus chilensis* | | captive | | KJ400228 | KJ400277 | KJ400306 | KJ400249 | KJ400289 |
| *Phoenicoparrus minor* | | captive | | – | KJ400278 | KJ400307 | – | KJ400290 |
|  |  | captive | | KJ400229 | KJ400279 | KJ400308 | KJ400250 | KJ400291 |
| *Phoenicoparrus andinus* | | captive | | KJ400230 | – | KJ400309 | KJ400251 | KJ400292 |
| *Phoenicoparrus jamesi* | | captive | | KJ400231 | KJ400280 | KJ400310 | KJ400252 | KJ400293 |
